# Forced Oscillation Detection via a Hybrid Network of a Spiking Recurrent Neural Network and LSTM

**DOI:** 10.3390/s25082607

**Published:** 2025-04-20

**Authors:** Xiaomei Yang, Jinfei Wang, Xingrui Huang, Yang Wang, Xianyong Xiao

**Affiliations:** College of Electrical Engineering, Sichuan University, Chengdu 610065, China; yangxiaomei@scu.edu.cn (X.Y.); wangjinfei@stu.scu.edu.cn (J.W.); xingruihuang@stu.scu.edu.cn (X.H.); fwang@scu.edu.cn (Y.W.)

**Keywords:** forced oscillation, natural oscillation, spiking recurrent neural network, long short-term memory, hybrid network

## Abstract

The detection of forced oscillations, especially distinguishing them from natural oscillations, has emerged as a major concern in power system stability monitoring. Deep learning (DL) holds significant potential for detecting forced oscillations correctly. However, existing artificial neural networks (ANNs) face challenges when employed in edge devices for timely detection due to their inherent complex computations and high power consumption. This paper proposes a novel hybrid network that integrates a spiking recurrent neural network (SRNN) with long short-term memory (LSTM). The SRNN achieves computational and energy efficiency, while the integration with LSTM is conducive to effectively capturing temporal dependencies in time-series oscillation data. The proposed hybrid network is trained using the backpropagation-through-time (BPTT) optimization algorithm, with adjustments made to address the discontinuous gradient in the SRNN. We evaluate our proposed model on both simulated and real-world oscillation datasets. Overall, the experimental results demonstrate that the proposed model can achieve higher accuracy and superior performance in distinguishing forced oscillations from natural oscillations, even in the presence of strong noise, compared to pure LSTM and other SRNN-related models.

## 1. Introduction

Detecting forced oscillations plays a significant role in ensuring the security and stability of power systems. Forced oscillations can cause undesirable power vibrations, damage equipment, and even interrupt the power supply. If the frequency of a forced oscillation is close to that of a poorly damped natural oscillation in power systems, it can lead to highly magnified energy. This can cause the forced oscillation to propagate across the entire system, potentially leading to catastrophic cascading blackouts [1,2,3].

Unlike natural oscillations caused by intrinsic natural interactions among dynamic components within the power system, forced oscillations are usually excited by periodic external disturbances, e.g., malfunctions in generator exciters, inverter controllers of renewable sources, and large-scale cyclic loads [1,2]. Therefore, the strategy of mitigating forced oscillations is different from that of natural ones; natural oscillations can be remedied by improving damping, while forced oscillations are effectively mitigated by removing forced oscillation sources [3]. To determine the correct remedial strategy, it is a prerequisite to distinguish forced and natural oscillations accurately.

With the help of synchrophasor data provided by phasor measurement units (PMUs), many methods of detecting forced oscillations or distinguishing them from natural oscillations have been developed. Theses methods are broadly divided into two categories: the traditional approaches and the deep learning approaches. The traditional methods commonly utilize spectral analysis and time–frequency representation to extract features of different oscillation signals [4,5,6,7,8,9,10,11,12]. For example, based on the mathematical representation of two oscillation types, a spectral approach is proposed using the noise responses and their spectral differences [8]. Based on statistical signatures from power spectral density, an oscillation diagnosis algorithm is developed [10]. To improve the detection performance when the frequency of forced oscillations is close to that of natural oscillations, a residual spectral analysis method is proposed [11]. To handle non-stationary oscillating signals, some time–frequency representation methods, such as short-time Fourier transform (STFT), STFT-based synchrosqueezing transform, and continuous wavelet transform, are utilized to extract non-stationary components, and then the time–frequency ridges with maximum energy are applied to detection the forced oscillations [6,9,13]. Further, some machine learning methods, such as the support vector machine (SVM) method and K-nearest neighbors algorithm, utilize the extracted features to distinguish two oscillation types [12] or to classify time-series data corresponding to the location of the forced oscillation sources [14]. Although traditional methods are computationally efficient and require fewer data, their heavy reliance on expert knowledge to extract features from oscillation signals limits their ability to handle the complex and changing operating conditions in power systems.

Recently, the success of deep learning methods has facilitated their application in the oscillation problems due to the strong feature extraction capability of these methods. Commonly, deep learning methods are mainly implemented based on artificial neural networks (ANNs), namely, the second generation of neural networks (NNs). For example, a shallow NN, consisting of a two-layer feedforward network, is proposed to distinguish forced oscillation from natural oscillation [15]. A hierarchical deep learning NN, comprising three NNs, is developed to perform FO detection, identification, and suppression [16]. Several NNs, based on long short-term memory (LSTM) and a convolutional NN (CNN), are employed to predict the Low-Frequency modes of oscillations [17]. To improve the training efficiency of deep learning models, a two-stage deep transfer learning and CNN method is proposed to convert the forced oscillation location problem into an image recognition problem [18]. Additionally, a new transfer branch-level transformer-based deep learning approach is proposed to localize the forced oscillation source without requiring extensive training data [19]. These developments indicate that ANNs are a fundamental technology in handling forced oscillation problems.

Despite the effectiveness of previous work based on ANNs, their inherent computational complexity and high power consumption pose significant challenges for forced oscillation detection applications, especially in edge computing. Typically, forced oscillations occur randomly in power systems due to various factors, such as changing operating conditions and the integration of renewable energy. It is essential to detect the presence of forced oscillations in a timely manner at edge devices, which are deployed at critical nodes in the power system, before these oscillations spread widely throughout the entire power grid and cause catastrophic cascading blackouts. However, edge devices are often designed with relatively limited computational resources (e.g., memory and storage) and constrained power supplies. Thus, deploying ANNs that require complex computations and high power consumption becomes difficult on edge devices.

In contract, spiking neural networks (SNNs) [20,21,22] have been theoretically proved to achieve greater computational and energy efficiency than ANNs [23], thanks to the advantage of a bio-inspired spiking computation mechanism. As the third generation of neural networks, SNNs are designed to emulate the information processing in the brain. While the structure of SNNs is similar to that of ANNs, unlike ANNs, which use continuous activation values to transmit information between neurons, SNNs utilize discrete spikes to represent and transmit information by encoding and processing data as electrical pulses or spikes. Since energy is mostly consumed only when a spike event occurs, this spiking-driven computation strategy of SNNs provides attractive energy-saving benefits, making it possible to deploy detection algorithms on edge devices [24,25]. Currently, SNNs have found applications in diverse domains, including power transformer fault diagnosis [26], image recognition [27], and more. However, SNNs are typically constructed as shallow networks and face the issue of training difficulty due to the non-differentiable nature of brain-like neurons. Consequently, the accuracy of SNNs is usually lower than that of popular ANNs [28,29]. Brief descriptions of the advantages and disadvantages of traditional methods, ANNs, and SNNs are shown in Table 1.

To explore the advantages of ANNs and SNNs, the hybrid networks of integrating SNNs and ANNs, such as the SNN–CNN [28,30], have been used and proven effective in some applications. Unlike these existing hybrid networks, we propose a novel hybrid network that integrates a spiking recurrent neural network (SRNN) with LSTM. In this hybrid architecture, the SRNN is constructed using adaptive leak integrate-and-fire (ALIF) neurons with self-recurrency. The ALIF neurons enhance computational capability through an adaptive firing threshold, while the recurrent mechanism within ALIF facilitates the capture of temporal dependencies in time-series data. Meanwhile, the LSTM, as a type of recurrent ANN, enables selective information processing, which further captures long-term dependencies and avoids issues such as vanishing gradients. The proposed hybrid network is directly trained by adopting a Gaussian function as a surrogate for the non-differentiable active one of the ALIF neurons in the backpropagation-through-time (BPTT) algorithm [31]. Finally, the trained hybrid network is applied to PMU data to implement the detection of forced oscillations, mainly distinguishing them from natural oscillations. Our contributions are mainly summarized as follows:

- The proposed hybrid network not only leverages the energy-saving advantage of SRNNs but also improves the detection performance of forced oscillations since combining with the LSTM can better capture the time-dependent information of oscillation data.
- Different from the existing detection methods based on ANNs, the proposed hybrid network driven by spike events is more friendly for deploying in edge devices.
- The proposed hybrid network achieves more satisfactory performance for detecting forced oscillations on simulated and real-world measured PMU data compared with other schemes, even in resonance conditions and periodically non-sinusoid injected disturbances.

The rest of this paper is organized as follows. The proposed methodology, including the problem formulation of forced oscillation detection, the proposed hybrid network, and the training strategy, is presented in Section 2. The detection performance and visual results of the proposed hybrid network are demonstrated in Section 3. Conclusions and potential future work are addressed in Section 4.

## 2. Methodology

In this section, we first introduce the problem formulation for forced oscillation detection. Then, we propose a hybrid network that integrates an SRNN structure with LSTM to distinguish forced oscillations from natural oscillations in PMU data. To train the proposed hybrid network directly, the BPTT algorithm is utilized. This involves replacing the discontinuous gradient with a smooth gradient function, which addresses the non-differentiability of the activation function in the brain-inspired neurons of the SRNN structure. Finally, several evaluation indices are introduced to assess the detection performance of the proposed hybrid network.

### 2.1. The Problem Formulation of Forced Oscillation Detection

Under the condition of forced oscillations, a mathematical model of the power system can be represented as a set of nonlinear differential Equations [32], given by

(1) $$\begin{array}{ccc} \dot{x}\left(t\right) & = & f(x\left(t\right),d_{u}\left(t\right),t)\\ y(t) & = & g(x\left(t\right),d_{u}\left(t\right),t), \end{array}$$

where f(·) and g(·) are the nonlinear functions, $x\left(t\right)\in R^{n}$ is a state vector (e.g., variables of the rotor angle and generator speed), $\dot{x}\left(t\right)\in R^{n}$ is the derivative of *x* concerning given time *t*, $y\left(t\right)\in R^{m}$ is a vector of output (e.g., variables of the voltage magnitude and voltage angle), and $d_{u}\left(t\right)\in R^{n_{j}}$ is a vector of periodic external disturbances with the number of $n_{j}$.

Due to the existence of periodic forced disturbances, there are sustained oscillations of the measurements (e.g., active power, voltage, and current) in the power system. Generally, forced oscillations interact with multiple natural modes, and the measured output vector y(t) can be modeled as [9,16]

(2) $$\begin{array}{ccc} y(t) & = & y^{N}\left(t\right)+y^{F}\left(t\right)+\epsilon \left(t\right)=\sum_{i=1}^{n_{i}}A_{i}^{N}e^{-\alpha_{i}^{N}t}\sin\left(\omega_{i}^{N}t\right)\\ & & +\sum_{j=1}^{n_{j}}A_{j}^{F}\sin\left(\omega_{j}^{F}t+\psi_{j}^{F}\right)+\epsilon \left(t\right) \end{array}$$

where $y^{N}\left(t\right)$ and $y^{F}\left(t\right)$ are the output vectors of dominant natural modes and forced oscillations, respectively. ($A_{i}^{N}$, $\alpha_{i}^{N}$,$\omega_{i}^{N}$) are the amplitude, damping ratio, and frequency of the dominant natural modes, respectively, ($A_{j}^{F}$, $\omega_{j}^{F}$, $\psi_{j}^{F}$) are the amplitude, frequency, and phase of the frequency components of forced oscillations, and $n_{i}$ and $n_{j}$ are the total number of natural modes and forced oscillations, respectively. ϵ(t) is measured noise.

The main target of detecting forced oscillations is to distinguish forced and natural oscillations by analyzing PMU data, whereas from (2), it can be observed that under forced oscillation conditions, the measured outputs y(t) contain $y^{N}\left(t\right)$, $y^{F}\left(t\right)$, and noise. In addition, when the frequency $\omega_{j}^{F}$ of the disturbance $u_{j}\left(t\right)$ is close to the frequency $\omega_{i}^{N}$ of the natural mode with a weak damping ratio, i.e., $\omega_{j}^{F}\approx \omega_{i}^{N}$, resonance phenomena occur, leading to a similarity with pure natural oscillations in most cases [2]. Thus, the existence of nonlinear, natural oscillations and resonance brings difficulty for forced oscillation detection.

### 2.2. Architecture of Proposed Hybrid Network

**Figure 1:**
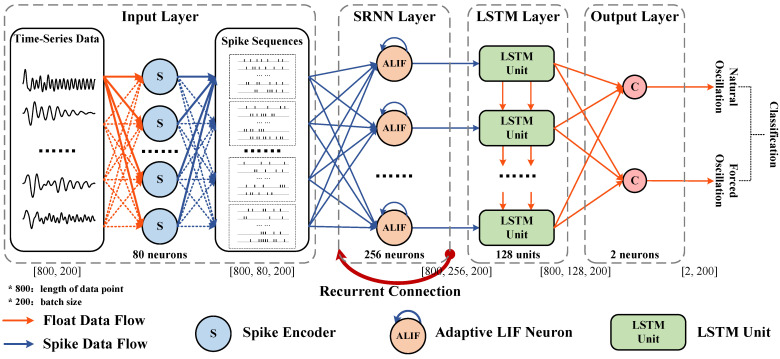
Structure of the proposed hybrid network.

To achieve both computational energy efficiency and high detection accuracy in detecting forced oscillations, a hybrid network integrating an SRNN structure with LSTM is proposed. The hybrid network mainly consists of four layers, i.e., one input layer, one SRNN layer, one LSTM layer, and one output classification layer. As shown in Figure 1, the input layer receives time-series float data and generates spike sequences through a spike encoder to be used as the input of the SRNN layer, and the SRNN layer is built with a set of ALIF neurons [33], as a spike response model to receive and dispose of time-dependent information. In addition, there are the self-recurrent connections of ALIF neurons within the SRNN layer and the lateral recurrent connections between the input layer and the SRNN layer. The LSTM layer is used to extract features related to more time-dependent information further, and the output layer provides the classification results to distinguish forced oscillations from natural oscillations. The dimensions of the input–output data flow in each layer are also shown in Figure 1. Moreover, the details of some key components within the proposed hybrid network are described as follows.

### 2.3. Spike Encoder

The spike encoder is utilized to convert the raw PMU time-series data within a time window into spike sequences, since the time-series data consist of continuous float-point real values, while the subsequent SRNN layer only accepts discrete spike trains. Before encoding time-series data into spikes, we normalize the values of data into the range of [0,1], adaptive to the measured data collected from different voltage levels. During the encoding process, a random number is generated with Poisson distribution or radial basic function (RBF), denoted as Poisson or RBF encoding in our experiments, and a spike event is triggered if the value of the generated random number is larger than the magnitude of the corresponding normalized input value [34,35]. In addition, an RBF-Th encoding, generating a spike when the generated random number is larger than a set threshold (Th) related to the input magnitude, is also utilized in Section 3.

**Figure 2:**
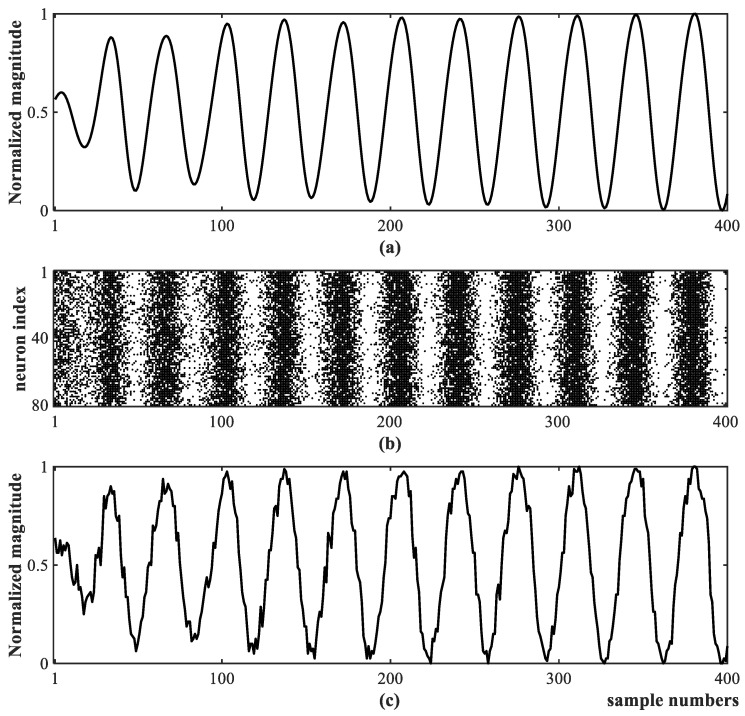
Generated spikes and their reconstructed signal using Poisson encoding: (**a**) forced oscillation raw PMU data; (**b**) spiking sequences; (**c**) reconstructed oscillation data from the spiking sequences in (**b**).

For a signal of forced oscillations, shown in Figure 2a, taking a Poisson encoding as an example, we show the spike sequences generated along time steps for 80 encoder neurons in Figure 2b, where the generated spikes are represented as black dots. The density of generated spikes at each time step corresponds to the value of the corresponding input data. To demonstrate the correctness of spiking encoding, we count the number of spikes for all 80 neurons at each time step to reconstruct the real number data and show the results in Figure 2c, similar to the original data shown in Figure 2a. The generated spiking sequence is used to simulate the biological realistic neurons for implementing forced oscillation detection tasks.

### 2.4. SRNN Layer with ALIF Neurons

**Figure 3:**
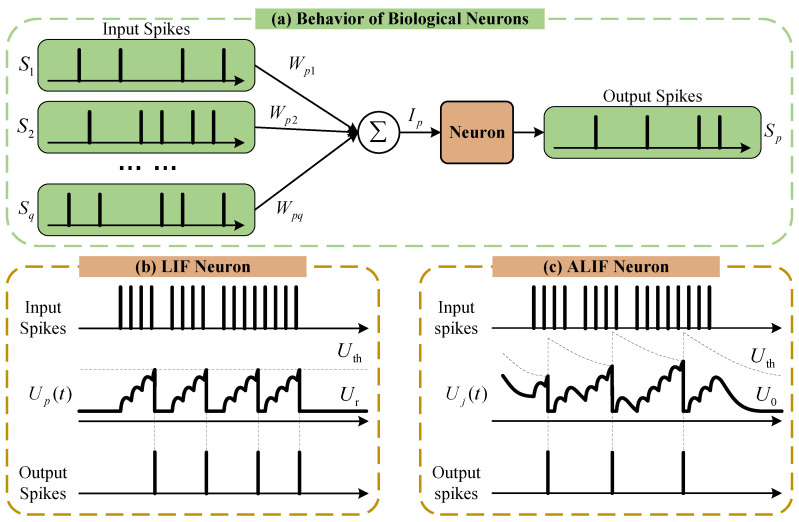
Spike generation of biological neurons. (**a**) Behavior of biological neuron; (**b**) LIF neuron; (**c**) ALIF neuron with adaptive threshold.

The SRNN layer is constructed from a set of ALIF neurons with self-recurrency, which emulate the behavior of biological neurons to receive and dispose of time-dependent information. The incoming spiking signals are integrated, and action potentials are fired when a certain potential threshold of the membrane is reached, as shown in Figure 3a. Different from the integrate-and-fire (LIF) neuron in Figure 3b commonly used in SNN structures, the ALIF neuron in Figure 3c, as a modified version of a LIF neuron, is used to improve the computation capability with an adaptive firing threshold in our works [33].

For a common LIF neuron *p*, the dynamic membrane potential (or voltage) $U_{p}\left(t\right)$ at time *t* is governed by a differential equation, given by

(3) $$\tau_{m}\frac{dU_{p}\left(t\right)}{dt}=-\left(U_{p}\left(t\right)-U_{r}\left(t\right)\right)+R_{m}I_{p}\left(t\right)$$

where $\tau_{m}$ and $R_{m}$ are the time constant and leaky resistance of the membrane, respectively, $U_{r}\left(t\right)$ is a resting membrane potential, and $I_{p}\left(t\right)$ is the input current of the neuron *p*, as shown in Figure 3a, given by

(4) $$I_{p}\left(t\right)=\sum_{q}W_{pq}S_{q}\left(t\right),$$

where $W_{pq}$ is the weight from presynaptic neurons *q* to the neuron *p*, and $S_{q}\left(t\right)$ are the incoming spikes from the presynaptic neurons *q*. When $U_{p}\left(t\right)$ reaches a certain threshold $U_{\mathrm{th}}$, the neuron *p* emits a spike, and $U_{p}\left(t\right)$ is reset to $U_{r}\left(t\right)$, as shown in Figure 3b. The process of firing and resetting in discrete time can be modeled as

(5) $$S_{p}\left(t\right)=\left\{\begin{array}{cc} 1 & \;\;\;\;\;\;U_{p}\left(t\right)\geq U_{\mathrm{th}}\\ 0 & \;\;\;\;\;\;U_{p}\left(t\right)<U_{\mathrm{th}} \end{array}\right.$$

and

(6) $$U_{p}\left(t+\delta t\right)=U_{p}\left(t\right)\left(1-S_{p}\left(t\right)\right)+U_{r}\left(t\right)S_{p}\left(t\right),$$

respectively, where δt is the minimum time step.

Although having computational simplicity, the LIF neuron lacks much of the more complex behavior of real neurons, e.g., the response for longer history dependencies. For this issue, the ALIF neuron augments an adaptive threshold, which increases upon neuron firing after each emitted spike and decays exponentially to a baseline threshold $U_{0}$, as shown in Figure 3c. For an ALIF neuron *j*, its threshold adaptation can be modeled as

(7) $$\begin{array}{ccc} U_{j,\mathrm{th}}\left(t\right) & = & U_{0}+\beta \eta_{j}\left(t\right)\\ \eta_{j}\left(t+\delta t\right) & = & \rho_{j}\eta_{j}\left(t\right)+\left(1-\rho_{j}\right)S_{j}\left(t\right)\\ \rho_{j} & = & \exp(-\delta t/\tau_{j,adp}), \end{array}$$

where β is a constant that controls the deviation $\eta_{j}$ from the baseline $U_{0}$, and $\rho_{j}$ is a parameter related to temporal dynamics, governing the exponential decay of the threshold with a time constant $\tau_{j,adp}$. With the adaptive threshold, the neural dynamics of ALIF neuron *j* in discrete time is given by

(8) $$U_{j}\left(t+\delta t\right)=\alpha_{j}U_{j}\left(t\right)+\left(1-\alpha_{j}\right)RI_{j}\left(t\right)-U_{j,\mathrm{th}}\left(t\right)S_{j}\left(t\right),$$

where $\alpha_{j}=\exp\left(-\delta t/\tau_{j,m}\right)$ represents the exponential decay of the membrane potential. Commonly, the behavior of ALIF neurons can be modeled as being self-recurrent with weights $\alpha_{j}$ and $\rho_{j}$, and the related self-recurrent parameters ($\tau_{j,m}$, $\tau_{j,adp}$) and weights $W_{pq}$ are trainable, expected to improve the performance of ALIF neurons [33].

### 2.5. Spike-Driven LSTM Layer

To further mine the temporal dependencies of spikes from the SRNN layer, an LSTM layer [36] is utilized in this model. As a special variant of recurrent neural networks (RNNs), LSTM not only inherits the ability of RNNs to capture the temporal dependencies of their inputs but also solves the problem of vanishing/exploding gradients when using the BPTT algorithm to learn from long-term sequences.

**Figure 4:**
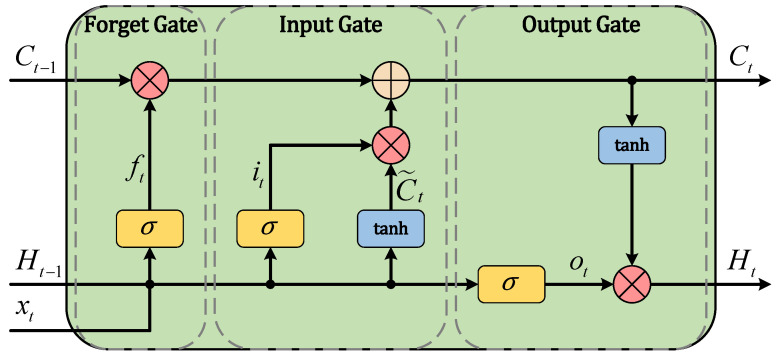
Structure of LSTM.

The spike-driven LSTM is similar to the conventional LSTM in terms of structure but different in terms of input state, which comprises spikes. Figure 4 shows the structure of the spike-drive LSTM unit, where $X_{t}$ is an input spike state from the previous SRNN layer, $f_{t}$ is a forget gate controlling how much of the previous state is transferred into the next state, $i_{t}$ is an input gate controlling the extent to which new data values are allowed to change the cell state, ${\tilde{C}}_{t}$ is the prospective new cell state, $o_{t}$ is an output gate controlling the part of the learned state returned by the model, $C_{t}$ is a cell state serving as the long-term memory, and $H_{t}$ is a hidden state made available to the memory. Concretely, the aforementioned elements such as data states and gates can be described by

(9) $$\begin{array}{ccc} f_{t} & = & \sigma (W_{xf}X_{t}+W_{hf}\;H_{t-1}+b_{f})\\ i_{t} & = & \sigma (W_{xi}X_{t}+W_{hi}H_{t-1}+b_{i})\\ {\tilde{C}}_{t} & = & \tanh(W_{xc}X_{t}+W_{hc}H_{t-1}+b_{c})\\ o_{t} & = & \sigma (W_{xf}X_{t}+W_{ho}H_{t-1}+b_{o})\\ C_{t} & = & f_{t}\times C_{t-1}+i_{t}\times {\tilde{C}}_{t}\\ H_{t} & = & o_{t}\times \tanh\left(C_{t}\right), \end{array}$$

where $C_{t-1}$ and $H_{t-1}$ denote the cell state and hidden states from the previous recurrent unit, σ denotes the sigmoid activation function, $W_{*}$ and $b_{*}$, i.e., $\theta_{LSTM}=\left(W_{*},b_{*}\right)$, denote weights and biases, respectively, that are both needed for training, and a symbol × denotes the Hadamard product.

### 2.6. Output Layer

A fully connected linear layer with 2 neurons is employed on the results from the previous LSTM layer, where 2 neurons correspond to the number of classes n=2, that is, forced oscillations and natural oscillations. Then, the softmax active function is used to predict the oscillation classes *z*, given by

(10) $$z={\mathrm{argmax}}_{z}\mathrm{softmax}\left(P\right),$$

where *P* denotes a predict vector consisting of a component $p_{i}$ from each class.

### 2.7. Training of the Proposed Hybrid Network

To train the hybrid network, the correct label of the time sequence is compared with the output of the whole hybrid network. Since the detection of forced oscillation is considered the classification task in our work, the cross-entropy function is used as a loss function Loss, given by

(11) $$L_{tot}\left(z_{i},{\hat{z}}_{i}\right)=\frac{1}{M}\sum_{i=1}^{M}\left[z_{i}\log\left({\hat{z}}_{i}\right)+\left(1-z_{i}\right)(1-\log\left({\hat{z}}_{i}\right)\right]$$

where *M* is the number of training examples, $z_{i}\in \left\{0,1\right\}$ is a target label for a training example $y_{i}$ in (2), measured by PMUs, and ${\hat{z}}_{i}=P_{\theta }\left(y_{i}\right)$ is the output probability generated by the proposed model with neural network parameters θ on the given $y_{i}$.

To update network parameters θ during the training process, the BPTT algorithm [31] is utilized to minimize the loss function in (11) by computing the partial gradient $\partial L_{tot}/\partial \theta$ with the chain rule. However, since the active function of the ALIF neuron in (5) is non-differentiable, the computation of the chain rule becomes difficult. For this issue, an alternative way is to replace the discontinuous gradient with a smooth gradient function. In our work, a Gaussian function $\left(G_{s}\left(\cdot \right)\right)$ is used as a surrogate gradient function for (5) during the backpropagation process, given by

(12) $$G_{s}\left(U_{p}\left(t\right)\right)=N\left(U_{p}\left(t\right)|U_{\mathrm{th}},\sigma_{g}^{2}\right)$$

where $\sigma_{g}$ is the standard deviation of the Gaussian. It was proven that the surrogate gradient resembles the actual gradient of the spikes [31], and the learnable parameters can be processed and updated by the BPTT algorithm with an Adam optimizer.

Given the loss function $L_{tot}$, the BPTT algorithm updates network parameters θ by computing the partial derivative $\partial L_{tot}/\partial \theta$. The learnable parameters $\theta_{LSTM}$ of the LSTM layer are updated as follows:

(13) $$\theta_{LSTM}^{k}=\theta_{LSTM}^{k}-\eta \frac{\partial L_{tot}}{\partial \theta_{LSTM}^{k}},$$

and the parameters of the SRNN layer, i.e., *W* in (4), $\tau_{adp}$ in (7), and $\tau_{m}$ in (8), are updated as

(14) $$\begin{array}{c} W^{k}=W^{k}-\eta \frac{\partial L_{tot}}{\partial W^{k}},\\ \tau_{adp}^{k}=\tau_{adp}^{k}-\eta \frac{\partial L_{tot}}{\partial \tau_{adp}^{k}},\\ \tau_{m}^{k}=\tau_{m}^{k}-\eta \frac{\partial L_{tot}}{\partial \tau_{m}^{k}}. \end{array}$$

where η∈(0,1) is the learning rate. Thus, the above learnable parameters can be implemented using the Adam optimizer, and the entire training procedure is outlined in Algorithm 1.
**Algorithm 1** The training procedure of the proposed SRNN-LSTM network.**Input:** 
Time-sequence data and the class label (*y*, *z*);**output:** 
Parameters of the SRNN-LSTM network;Initialize: randomly initialize parameters of SRNN-LSTM, epochs, and learningrateη;Preprocessing: data segmentation and normalization.Encoding: s = SpikingEncoding(y);**for** epoch=1 to epochs **do**   % forward propagation process   x = SRNN(s);   p = Softmax(LSTM(x));   $\hat{z}=\max\left(p\right)$;   % back propagation process   Compute $L_{tot}\left(z,\hat{z}\right)$;   $\theta_{LSTM}\leftarrow \theta_{LSTM}-\eta \frac{\partial L_{tot}}{\partial \theta_{LSTM}}$;   $W\leftarrow W-\eta \frac{\partial L_{tot}}{\partial W}$;   $\tau_{adp}\leftarrow \tau_{adp}-\eta \frac{\partial L_{tot}}{\partial \tau_{adp}}$;   $\tau_{m}\leftarrow \tau_{m}-\eta \frac{\partial L_{tot}}{\partial \tau_{m}}$**end for**

### 2.8. Evaluation Metrics

To evaluate the detection performance of the proposed hybrid network, several evaluation indices, i.e., Accuracy (Acc), Precision (Pre), Recall (Rec), and F1-score (F1), are used. The formulas of the four indices are calculated as

(15) $$\begin{array}{ccc} \; & \mathrm{Acc}=\frac{TP+TN}{TP+TN+FP+TN} & \mathrm{Pre}=\frac{TP}{TP+FP}\\ \; & \mathrm{Rec}=\frac{TP}{TP+FN} & F1=\frac{2*\mathrm{Pre}*\mathrm{Rec}}{\mathrm{Prec}+\mathrm{Rec}}, \end{array}$$

where TP, FN, TN, and FN are the number of true positives, false positives, true negatives, and false negatives, respectively.

## 3. Experimental Results and Discussion

In this section, the performance of the proposed hybrid network is analyzed and compared with LSTM and two SRNN-related models (i.e., SRNN + SRNN and SRNN + SRNN + LSTM) using both simulated and real-world data. The LSTM is used as the representative of ANNs. The SRNN + SRNN network is employed to assess the impact of integrating LSTM with an SRNN, while the SRNN + SRNN + LSTM network is utilized to evaluate the performance of adding more SRNN layers, particularly in scenarios with limited training data. To intuitively demonstrate the effectiveness of the proposed model, visualization analyses are conducted for the simulated oscillation data under two special operating scenarios: the resonance case and periodically non-sinusoidal injected disturbances. Additionally, experiments on simulated data are carried out to analyze the impact of different parameters on the proposed model, mainly including β and $U_{0}$ in (7), and $R_{m}$ in (3).

### 3.1. Datasets

**Figure 5:**
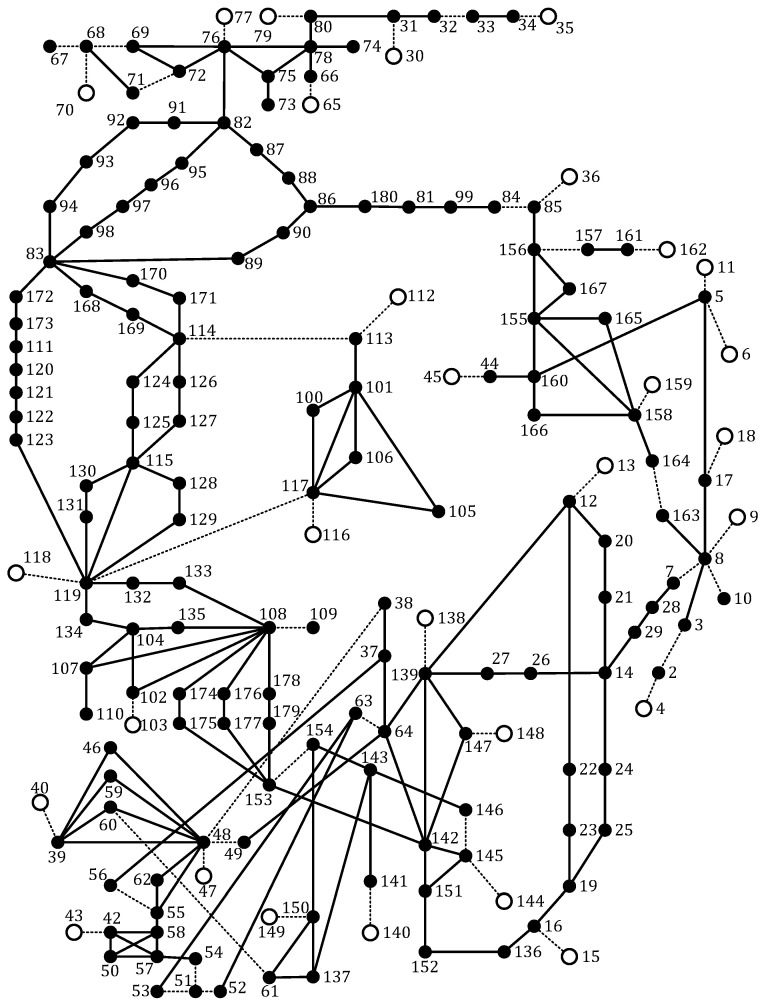
WECC 179-bus model as the test system.

Simulated and real-world measured PMU data are utilized in our experiments. The simulated PMU data are generated through 40 s time-domain simulations using TSAT 10.0 software on a WECC 179-bus system with 29 machines, as shown in Figure 5 [37]. There are 27 simulated oscillation cases, i.e., 9 poorly damped natural electromechanical oscillation cases and 18 forced oscillation cases. The natural oscillation cases include a set of combining the following scenarios, e.g., local modes or inter-area modes, or a combination of both generated from single or multiple sources, while the forced oscillation cases include resonance or non-resonance scenarios. The total number of simulation data is 14,202 time-sequence samples. Meanwhile, the real-world PMU measured data have 1327 time-sequence samples and consist of six actual oscillation events, captured in an ISO New England (ISO-NE) system [38].

To enhance the dataset, we employ a sliding window segmentation approach. Each signal sample is configured with a length of 800×1 data points, and the values of the data are normalize into the range of [0, 1], adapting to the measured data collected from different voltage levels. From the simulated and/or real-world data, we randomly selected 75% of samples for training and 25% of samples for testing, i.e., a ratio of 4:1.

### 3.2. Parameter Setting

The proposed model is implemented in Python 3.10 and Pytorch 2.1.2 and is trained on a 13th Gen Intel (R) Core (TM) 15-13490F CPU and NVIDIA GeForce RTX 3060 GPU. There are 80 encoding neurons in the input layer, 256 ALIF neurons in the SRNN layer, 128 units in the LSTM layer, and 2 neurons in the output layer, as shown in Table 2. The main parameters of the ALIF neurons and key hyperparameters of network training are shown in Table 3.

The number of neurons in the other three comparative networks is also shown in Table 2. For a fair comparison, the LSTM network has the same number of layers and the same number of neurons in each layer as the proposed model. The SRNN + SRNN network is constructed with only two SRNN layers, without an LSTM layer, while the SRNN + SRNN + LSTM network is constructed with two SRNN layers and one LSTM layer. Three encoding methods, i.e., Poisson, RBF, and RBF-Th, are utilized to generate spike sequences for the proposed model and the two SRNN-based networks. Moreover, the parameters of the ALIF neurons and network training are kept the same for the three SRNN-based networks.

### 3.3. Results with Simulation Data

**Figure 6:**
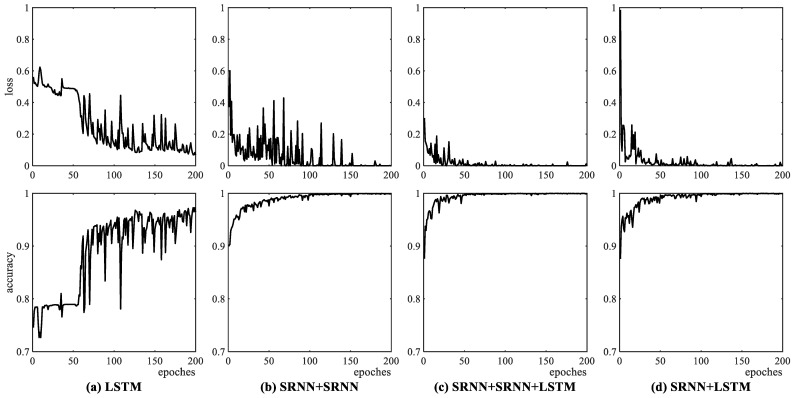
Loss and detection accuracy curves versus epochs for four networks during training procedure. (**a**) LSTM; (**b**) SRNN + SRNN; (**c**) SRNN + SRNN + LSTM; (**d**) proposed SRNN + LSTM.

The simulation data are first used to train and test the performance of the four networks, where the three SRNN-related models utilize three different encoding methods. Figure 6 shows the loss and accuracy curves of the four models during the training process, with RBF encoding applied to the three SRNN-related models. As can be seen from Figure 6, the LSTM converges at a slower rate and experiences larger fluctuations in both loss and accuracy curves compared to the other three SRNN networks. The reason is that multiple LSTM layers are susceptible to the exploding gradient problem, which causes the weights to update by large amounts, leading to instability of the loss function. In addition, comparing among the three SRNN-related models, the loss values of the two models with an LSTM layer decrease in stability faster than that of the SRNN + SRNN model without an LSTM layer, and the two models with an LSTM layer achieve detection accuracy values exceeding 97% after an epoch of 50 during the training procedure. This indicates that combining SRNN and LSTM layers is conducive to the training of the SRNN-based model. Using other encoding methods, the curves of loss and accuracy from the three SRNN-related models are similar to those of RBF encoding.

The four trained networks are utilized to test other simulation data, which are not interlaced with the training data. The evaluation indices in (15) are calculated and listed in Table 4. As shown in Table 4, the LSTM achieves high precision and low recall. This implies that the LSTM is good at correctly detecting forced oscillation cases but misses many actual forced oscillation instances that exist in the data. Thus, the accuracy and F1 score of the LSTM are not satisfactory due to the limited training epochs and data. Meanwhile, the two SRNN-related models with an LSTM layer can provide higher values in each index compared to the SRNN + SRNN model without an LSTM layer. This demonstrates that integrating an SRNN with LSTM can provide satisfactory detection performance. Moreover, the proposed SRNN + LSTM network achieves the highest accuracy and precision among the four networks in the case of RBF or RBF-Th encoding.

### 3.4. Results with Addition of Real-World Data

In this experiment, four networks are re-trained using data that include both simulation data and limited real-world measurement data to evaluate their performance in real operating scenarios. The training and testing settings are the same as those in Section 3.3. The performance indices for each model are shown in Table 5.

Compared with Table 4, the indices for each network in Table 5 decrease due to the introduction of measurement data. The reason is that the data distribution of the measurement data exhibits differences from that of the simulation data. Moreover, the amount of measurement data is less than that of the simulated data. This causes the degraded performance of the trained network on the testing data, which are randomly selected partly from the measurement data and partly from the simulation data. Moreover, in the case of Poisson encoding, integrating LSTM with an SRNN layer causes the detection failure. The reason for this phenomenon needs to be explored in future work.

We also note that although the LSTM has a certain level of detection accuracy, its recall and F1 score are quite low. This is because the LSTM misclassifies a significant number of forced oscillations as natural oscillations. Since the number of correctly detected natural oscillation samples is high, the overall accuracy of the LSTM appears to be satisfactory. However, integrating LSTM and an SRNN is helpful in effectively mining temporal dependencies in time-series oscillation data and achieves good performance. In addition, comparing the SRNN + SRNN + LSTM network with the proposed SRNN + LSTM network, the indices in Table 5 also show that adding more SRNN layers cannot bring about an improvement of detection performance under limited data. Overall, the proposed hybrid network indicates its classification performance for forced oscillations and natural oscillations.

### 3.5. Visual Analysis

To visualize the operation process of the proposed hybrid network, taking a resonance case and periodically rectangular injected signal case as examples, we present the fired spikes of input and SRNN layers and prediction results after the LSTM and output layers in this section.

**Figure 7:**
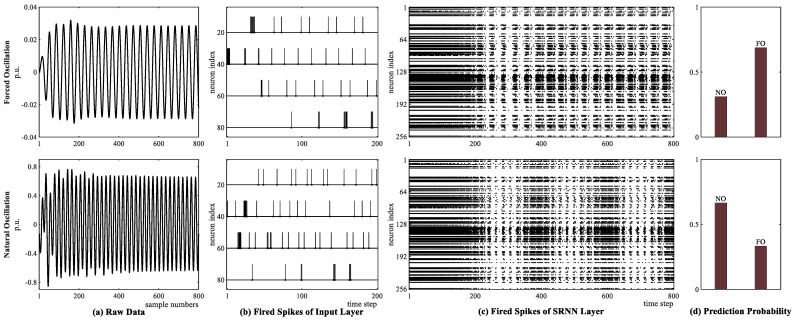
For the case of resonance, the results of three key layers in the proposed hybrid network. The first row refers to the forced oscillation, which resonates with local natural oscillation; the second row refers to the natural oscillation. (**a**) PMU voltage raw data; (**b**) fired spikes generated by RBF encoding in the input layer; (**c**) fired spikes of the SRNN layer; (**d**) prediction probability after LSTM layer and softmax function.

First, a resonance case is considered, where forced oscillation is caused by the sinusoidal signal injected at generator 4 in Figure 5 using exactly the local natural mode frequency of 0.86 Hz. To demonstrate that the proposed hybrid network is adapted to voltage signals with weak oscillation signatures, we take the voltage of forced oscillation far away from the oscillation resource as an example. The shape of voltage data is similar to that of natural oscillation, as shown in Figure 7a. The resonance makes it difficult to determine whether the forced oscillation exists. Two voltage data are, respectively, input into the proposed hybrid network, and the pulse spikes are generated by the RBF-based spiking encoder in the input layer.

To observe the generated spikes clearly, only spikes from four neurons, i.e., 20, 40, 60, and 80, within the range of time step [1, 200] are shown in Figure 7b for the forced and natural oscillations, respectively. After the pulse spikes are processed by ALIF neurons in the SRNN layer, the fired spikes of each ALIF neuron at each time step are represented by a single bit in the block dot, as shown in Figure 7c. Each column represents the fired spike results for 256 ALIF neurons at each time step, and each row represents the fired spikes of a certain ALIF neuron at 800 time steps. The successive spikes from time 1 to 200 indicate an increasing trend in the signal value, while the absence of spikes signifies a decrease in the signal. Meanwhile, the intervals between dense spikes and sparse spikes also reflect the frequency of the signal. After the SRNN layer, the fired spikes are fed into the LSTM layer and classification layer; the prediction probability results for two types of oscillation are shown in Figure 7d, respectively. The spike differences in the input and SRNN layers between two oscillation signals demonstrate that the proposed hybrid network can effectively detect forced oscillations in resonance cases.

**Figure 8:**
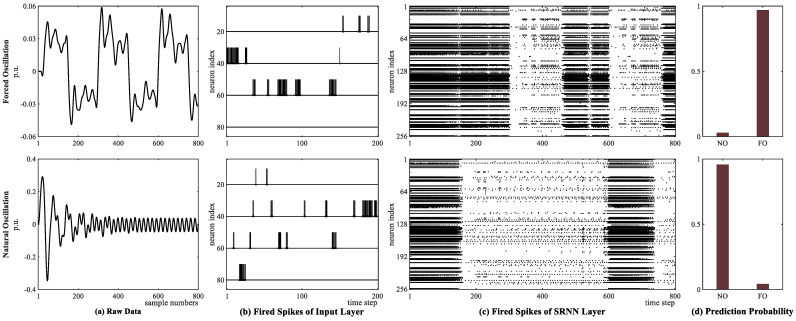
For the case of periodic/rectangular injected disturbance, the results of three key layers in the proposed hybrid network. The first row refers to forced oscillation; the second row refers to the natural oscillation. (**a**) PMU voltage raw data; (**b**) fired spikes generated by RBF encoding in the input layer; (**c**) fired spikes of the SRNN layer; (**d**) prediction probability after LSTM layer and softmax function.

Next, the other oscillation case, caused by a periodically rectangular disturbance signal, is considered. The voltage signal of the forced oscillations shows the tendency of rectangular shape as shown in Figure 8a. Accordingly, to show the detection effectiveness of the proposed hybrid network, we choose voltage data of the natural oscillation shown in Figure 8a, whose initial stage is similar to that of forced oscillation, as an example. Similarly, the processing results from the input layer using RBF-based encoding, the SRNN layer, and the LSTM + softmax layer for two types of oscillations are shown in Figure 8b–d, respectively. We also note that the density distribution of the fired neurons in Figure 8c is different from that in Figure 7c. This difference is attributed to the distinct responses of the encoding and firing mechanisms to rectangular and sinusoidal signals. From Figure 8, it can be observed that the proposed hybrid network can effectively distinguish forced oscillations from natural oscillations in a periodically rectangular injected disturbance signal since there are obvious spike differences in the input and SRNN layers between the two oscillation types.

### 3.6. Discussion

#### 3.6.1. Parameter Sensitivity

To analyze the parameter sensitivity of the proposed SRNN-LSTM hybrid model, we conduct experiments on simulation data with the RBF-th encoding strategy. The parameters considered include β and $U_{0}$ in (7) and $R_{m}$ in (3). These parameters were chosen because β and $U_{0}$ are key parameters for the adaptive firing threshold of the ALIF neuron and $R_{m}$ is related to dynamic membrane potential (or voltage).

For β, we examined values ranging from 0.0 to 3.0 in intervals of 0.6. Here, β=0 indicates that a non-adaptive threshold is used. When β is varied, the other parameters are kept constant as specified in Section 3.2. As shown in Table 6, the detection accuracy of the model initially decreases with increasing β, reaches its highest point when β=1.8, and then decreases again. In short, an appropriate setting for β tends to yield better results; conversely, this is not the case. For $U_{0}$ and $R_{m}$, we considered ranges of [0.07, 0.12] and [0.4, 1.4] with intervals of 0.01 and 0.2, respectively. As their values increase, the detection accuracies of the model first increase, reach corresponding peaks, and then decrease. The underlying cause of this phenomenon remains to be explored in our future work.

#### 3.6.2. Robustness on Various Noise Level

To demonstrate the noise robustness of the proposed hybrid network, we compare its detection accuracy with three other comparative networks. The simulation data are mixed with noise at SNRs ranging from +50 to 0 dB for both training and testing. The noisy float-point data are then converted into spike sequences using the RBF-Th encoding method for the three SRNN-related models. After training and testing the four models on the noisy simulation data, we collect the prediction results and present the corresponding accuracy values in Table 7.

The proposed hybrid network achieves an accuracy of 98.83–93.06% across the SNR levels of 50 to 0 dB, although the accuracy decreases as the noise increases, which is the same as in the other three comparative models. However, as shown in Table 7, the accuracy decline of the proposed SRNN-LSTM network is largely lower than that of the other methods. The SRNN + SRNN + LSTM and the proposed model, which integrates LSTM with SRNN, show much smaller accuracy drops than pure LSTM and SRNN models. These results highlight that the integration of an SRNN and LSTM enhances the noise robustness of the model.

## 4. Conclusions

This paper proposes a hybrid network that combines an SRNN with an LSTM structure to distinguish forced oscillations from natural oscillations. For oscillation PMU data, the time-series float-point data are first converted into spiking sequences using a spiking encoder. The SRNN leverages the advantages of ALIF neurons with self-recurrence to enhance computational capability while inheriting the inherent high energy efficiency of the spiking computation mechanism. Integrating the SRNN with the LSTM structure further enhances the capability of mining temporal dependencies in spike signals. Extensive experiments were conducted on both simulated and limited real-world PMU data to verify the effectiveness of the proposed model. The experimental results demonstrate the superiority of the proposed hybrid model over pure LSTM and other SRNN-related models for detecting force oscillations. In a nutshell, the proposed model reaches a satisfactory detection performance, even in the presence of strong noise.

In the future, the performance of the proposed model can be further improved. For instance, the results obtained using limited real measured data show that the performance of the proposed model is not as high as that using simulation data. The small sample size of real-world data is one of the main reasons for the performance degradation. Thus, how to train the proposed model effectively for small-size data needs to be addressed in future work. Moreover, the underlying causes of the proposed model’s parameter sensitivity remain to be explored in our future work. For real applications, the proposed model can be considered for implementation in FPGA and deployment in edge devices to detect forced oscillations in a timely manner. 

## Figures and Tables

**Table 1:** Comparison of advantages and disadvantages of traditional methods, ANNs, and SNNs.

Methods	Advantages	Disadvantages
Traditional	Low computational cost.	Feature extraction complexity.
	Easy to implement.	Limitation of handling complex
	Less data requirement.	and changing operating conditions.
ANNs	Strong feature extraction capability.	Inherent computational complexity.
	Effective networks.	High power consumption.
SNNs	Energy-efficient.	Non-differentiable.
	Bio-inspired.	Training difficulty.

**Table 2:** Number of neurons in each layer of three networks.

Network	Input Layer	Hidden Layers	Output Layer
LSTM	80	[256, 128]	2
SRNN + SRNN	80	[256, 128]	2
SRNN + SRNN + LSTM	80	[256, 128, 128]	2
Proposed (SRNN + LSMT)	80	[256, 128]	2

**Table 3:** Parameters of ALIF neurons and network training.

Parameters of ALIF	Values	Training Parameters	Values
$R_{m}$ in (3)	1	length of time window	800
β in (7)	1.8	batch size	200
$U_{0}$ in (7)	0.5	epoch	200
$\sigma_{g}$ in (12)	0.5	learning rate	$1\times 10^{-2}$

**Table 4:** Evaluation indices for four networks on simulation testing data.

Network	Encoding	Acc (%)	Pre (%)	Rec (%)	F1 (%)
LSTM	None	87.48	99.90	66.67	79.97
SRNN + SRNN	RBF	87.16	76.37	95.24	84.76
	RBF-Th	91.73	96.09	81.27	88.06
	Poisson	95.19	90.93	96.83	93.78
SRNN + SRNN + LSTM	RBF	94.52	98.98	87.93	93.13
	RBF-Th	93.88	96.53	86.79	91.41
	Poisson	95.45	92.25	95.47	93.83
Proposed (SRNN + LSMT)	RBF	95.61	92.02	96.70	94.30
	RBF-Th	98.83	99.80	97.08	98.42
	Poisson	94.19	91.56	93.08	92.32

**Table 5:** Evaluation indices for four networks with the addition of real-world measurement data into the simulation data.

Network	Encoding	Acc (%)	Pre (%)	Rec (%)	F1 (%)
LSTM	None	75.32	50.00	0.10	0.19
SRNN + SRNN	RBF	85.23	91.05	44.57	59.84
	RBF-Th	86.13	97.71	44.86	61.48
	Poisson	83.92	76.83	49.90	60.51
SRNN + SRNN + LSTM	RBF	84.76	92.22	41.81	57.53
	RBF-Th	86.78	96.74	48.10	64.25
	Poisson	xx	xx	xx	xx
Proposed (SRNN + LSMT)	RBF	87.18	98.09	49.05	65.39
	RBF-Th	86.74	89.32	52.57	66.18
	Poisson	xx	xx	xx	xx

xx: denotes detection failure.

**Table 6:** Accuracy under different parameter settings for the proposed hybrid network.

β	Acc (%)	$U_{0}$	Acc (%)	$R_{m}$	Acc (%)
**0.0**	95.33	**0.07**	95.57	**0.4**	93.55
**0.6**	93.36	**0.08**	95.60	**0.6**	94.50
**1.2**	91.17	**0.09**	97.48	**0.8**	96.57
**1.8**	98.83	**0.10**	98.83	**1.0**	98.83
**2.4**	95.83	**0.11**	96.62	**1.2**	95.12
**3.0**	93.26	**0.12**	95.29	**1.4**	93.60

**Table 7:** Detection accuracy of four networks under different SNR levels.

Network (Acc (%))	SNR (dB)						
	**0**	**10**	**20**	**30**	**40**	**50**	**Noise-Free**
LSTM	71.16	71.40	75.84	85.03	86.50	87.05	87.48
SRNN + SRNN	79.29	83.37	86.53	90.26	91.40	93.55	95.19
SRNN + SRNN + LSTM	85.44	87.75	91.48	92.16	93.95	95.23	95.45
Proposed (SRNN + LSTM)	93.06	94.69	97.01	97.98	98.83	98.83	98.83

## Data Availability

The data presented in this study are available from [38].

## Abbreviations

The following abbreviations are used in this manuscript:

SNN	Spiking neural network
SRNN	Spiking recurrent neural network
PMU	Phasor measurement unit
BPTT	Backpropagation through time
ALIF	Adaptive leak integrate-and-fire
RBF	Radial basic function
RBF-Th	Radial basic function with a set threshold
LIF	Leak integrate-and-fire
NO	Natural oscillation
FO	Forced oscillation
